# Accurate Monitoring and Fault Detection in Wind Measuring Devices through Wireless Sensor Networks

**DOI:** 10.3390/s141122140

**Published:** 2014-11-24

**Authors:** Komal Saifullah Khan, Muhammad Tariq

**Affiliations:** Department of Electrical Engineering, National University of Computer & Emerging Sciences (NUCES), Peshawar Campus, Peshawar 25000, Pakistan; E-Mail: tariq.khan@nu.edu.pk

**Keywords:** wind speed monitoring, error detection, wireless sensor networks, site assessment, cup anemometers

## Abstract

Many wind energy projects report poor performance as low as 60% of the predicted performance. The reason for this is poor resource assessment and the use of new untested technologies and systems in remote locations. Predictions about the potential of an area for wind energy projects (through simulated models) may vary from the actual potential of the area. Hence, introducing accurate site assessment techniques will lead to accurate predictions of energy production from a particular area. We solve this problem by installing a Wireless Sensor Network (WSN) to periodically analyze the data from anemometers installed in that area. After comparative analysis of the acquired data, the anemometers transmit their readings through a WSN to the sink node for analysis. The sink node uses an iterative algorithm which sequentially detects any faulty anemometer and passes the details of the fault to the central system or main station. We apply the proposed technique in simulation as well as in practical implementation and study its accuracy by comparing the simulation results with experimental results to analyze the variation in the results obtained from both simulation model and implemented model. Simulation results show that the algorithm indicates faulty anemometers with high accuracy and low false alarm rate when as many as 25% of the anemometers become faulty. Experimental analysis shows that anemometers incorporating this solution are better assessed and performance level of implemented projects is increased above 86% of the simulated models.

## 1. Introduction

Over the past few years energy production through wind has increased dramatically because of the wind's potential economic, environmental and security benefits. Wind energy resources provide cheaper and economical electricity as compared to other energy resources. In the US wind farms are generating electricity at a rate less than 5 cents per kilowatt [1]. Due to the fast increase in wind technology deployment, developing projects require much better and accurate monitoring along with supervising techniques. Very accurate wind speed measurements are very important to the wind energy community. According to [1], many installed projects report poor performance due to poor resource assessment and poor fault prediction techniques. Errors in wind speed measurement are one of the major reasons for causing uncertainties in power generation performance of wind turbines. Accurate monitoring of parameters (like wind speed, direction, overspeeding, number of rotations, torque, turbulence intensity and temperature) is therefore a major contribution to the wind energy community.

Wind measuring devices like anemometers are used for evaluating a potential site for wind energy projects. Regular maintenance and accurate monitoring of anemometers, installed for site assessment, is one of the main challenges for wind energy deployment companies because of the high costs required for it. Errors occurring in the readings taken by these devices are usually checked manually by sending labor to the area where these devices are installed. No proper monitoring techniques for these devices are present in order to get accurate measurements by these devices which in turn results in inaccurate assessment of the site. This badly affects the projects that are to be implemented in that area as the projects when implemented perform far less than expected. In our analysis and experimentation, a WSN will play a vital role in monitoring and supervising wind resources in remote areas and reporting the data back to the central control station. A number of different locations can be monitored through one station. This network will minimize the cost of cables and the labor that will be needed to manually check the sites and other costly accessories.

Cup anemometers are accepted by international standards for power performance measurements and are used widely in wind energy applications [2]. Deployments in remote areas require regular engagement and more oversight than conventional wind energy projects [3]. According to National Renewable Energy Laboratory (NREL) in the US, many wind energy projects report results as low as 60% of the predicted performance [1]. The reason for this is poor resource assessment and the use of new untested technologies and systems in remote locations. Hence, better assessment and fault detection techniques are required to achieve the desired performance level. For a balanced and economical system, it is important to reduce operational costs of the project. Condition-based maintenance systems will help in achieving accurate and improved performance in energy projects to be implemented and will decrease the operational costs. All wind turbines installed worldwide by the end of 2008 are generating 260 Terawatt hour (TWh) per annum, corresponding to more than 15% of the global energy consumption [4]. It is clear that in the mid- to long-term, wind energy investments will rather be strengthened due to their low risk character and additional economic benefits. The contributions of this paper are as follows:

- We are the first to introduce a WSN for accurate monitoring and fault detection in wind measuring devices. We have used our own developed sensor nodes instead of traditional sensor nodes such as MICAZ, TelosB, or IRIS, which does not suit our experiments due to limited radio range and sensing capabilities.
- This paper proposes a technique for accurate site assessment. Proper assessment of a site will lead to accurate estimations of the energy production from future wind energy projects to be implemented.

To the best of our knowledge, no work has been done yet on implementing a WSN for site assessment purposes. The remainder of the paper is organized as follows: Section 2 explains the related work in detail. Section 3 explains the design description and Section 4 explains the proposed algorithm. Section 5 demonstrates the simulation and experimental results. Finally we conclude the paper in Section 6.

## 2. Related Work

Some significant work that has been done on monitoring fault issues in anemometers is summarized below.

### 2.1. Fault Detection Techniques in Sensors and in Wind Measuring Devices

Good quality work has been done on detecting faults in sensors. In [5], failed nodes were traced at the base station, assuming that all sensor measurements will be directed to the base station through a routing tree. In this work, the base station has a global view of the network topology and it can identify failed nodes through route update messages. In recent works for fault detection [6–8], sensors are required to access a binary decision procedure by comparing their readings with a predetermined threshold. This binary decision is called a 0/1 decision predicate with 0 indicating a normal status and 1 indicating a faulty status.

Some research work has also been done in identifying faults in wind measuring devices. A technique for data analysis was discussed in [9] to identify faults in a wind turbine installed in a wind farm. The data analysis for this purpose was done through automated Supervisory Control and Data Acquisition (SCADA). In [10], an algorithm for fault detection in wind turbines was discussed. Condition monitoring techniques were introduced in order to detect faults in different components of the turbine. Sensors were also used for environmental monitoring applications. Also, for environmental conditions monitoring, a WSN deployment was carried out to monitor environmental conditions [11]. This technique relied on installing a low cost station in the alpines in Switzerland which was a better technique for environmental monitoring as compared to high cost stations installed for this purpose. In [12], an anemometer-based method for remote area wind energy harvesting was presented. The motion of the anemometer shaft was utilized to turn an alternator which in turn would be useful for harvesting energy.

In the past a lot of work has been done for developing wind measuring devices that will accurately measure wind speed. In 1990, a one dimensional, ionic flow anemometer was developed in Japan using a high voltage pulse [13]. The air molecules were ionized and the flow time detected for the ionized molecules was used for wind speed calculation. But by using this technique only single direction measurements could be made and the right direction was obtained by trial and error method. Another one dimensional, fixed electric field measurement technique was applied in 1995 [14]. Air molecules were ionized using high voltage between polar plates in a fixed distance. In 2009, a mobile optical coordinate measuring technology was used to acquire offshore wind turbine data and behaviors [15].

### 2.2. Site Assessment Techniques

Site assessment for wind energy projects can also be done with the use of wind maps. In 2011, a Geographic Information System (GIS)-based wind farm site assessment technique was used. Using this method, large geographic areas could be assessed and the evaluation outcome could be displayed in suitable maps [16]. Wind atlases are also used for site assessment. In [17], a wind resource atlas was prepared where wind observations were recorded from meteorological stations around that area. These recorded observations were used to define the statistical details of the wind resource in that particular area. However, as these maps short fall of giving accurate results, these are useful only for initial selection of sites. Although improvements have been made to obtain accurate results by these maps, it is still improbable that these maps will end the importance and need for on-site measurements for site selection. However, this technique can still help in speeding up the process for proper site selection. Apart from wind maps, Laser Imaging Detection and Ranging (LIDAR) and Sonic Detection and Ranging (SODAR) are also used for site assessment. SODAR and LIDAR are basically remote sensing (RS) devices that use sound and light, respectively. These technologies are ground-based and are capable of working for quite long distances which make them easy to use for analyzing and evaluating a site. In September 2013, wind speed data was collected at Buena Vista Wind Farms in California using a LIDAR instrument (Zephir 300) [18]. However, the LIDAR required more power than expected and measured high wind speeds at night. The reason for this was that the LIDAR was not placed on truly flat land. Hence, these ground based RS devices show variable and unpredictable measurements, especially in hilly terrains. Improvement of these devices is very important in order to increase the confidence in using these ground based techniques [19]. Up till now, wind site assessment process has counted on cup anemometers and approved it as a standard for this purpose [2].

## 3. Design Description

Before explaining the design details, we first give background information about the cup anemometers that will be used. Then we explain the comparison of anemometer with a potential RS technology, SODAR. After that we give the details of proposed design.

### 3.1. Background

An anemometer is a device that is used for measuring wind speed in any particular area. Typically, anemometers are deployed in an area for assessing the potential of a site for future wind energy projects. By using anemometers, we can analyze the following parameters:

#### 3.1.1. Wind Speed and Direction Analysis

For cup anemometer, we consider a three dimensional wind speed vector [20]. The 3 dimensional wind speed vector is given as:

(1) $$\vec{V}=(x,y,z)$$

where:

*x* = *Longitudinal Component*
*y* = *Traversal Component*
*z* = *Vertical Component*

Hence:

(2) $$\left|\vec{V}\right|=\int_{t}\sqrt{x^{2}+y^{2}+z^{2}}$$

In most cases, the vertical component is excluded which makes the equation as:

(3) $$\left|\vec{V}\right|=\int_{t}\sqrt{x^{2}+y^{2}}$$

The difference between these two wind speed values in Equations (2) and (3) is based on turbulence intensity (Ti). For 15% turbulence, the difference is 0.5% and for 30% turbulence the difference is 1% [20]. The difference increases with high turbulence. In our analysis, we use the three dimensional wind speed vector.

We will focus on the three dimensional component of wind for accuracy. So, according to [21], the instantaneous wind speed is decomposed into its components. We assume that mean wind speed is horizontal:

(4) $$\left\{\begin{array}{c} x\\ y\\ z \end{array}\right\}=\left\{\begin{array}{l} V_{f}+x\\ y\\ z \end{array}\right\}$$

where *V_f_* represents the fluctuations in wind speed. We assume that these fluctuations are small as compared to mean wind speed. The direction, θ for the three dimensional wind speed will become:

(5) $$\theta =\tan^{-1}\frac{z}{\sqrt{x^{2}+y^{2}}}$$

#### 3.1.2. Effect of Turbulence

Turbulence intensity is dependent on mean wind speed and standard deviation. Lower the wind speed, higher will be the turbulence intensity:

(6) $$T_{i}=\frac{\sigma }{\bar{V}}$$

where, σ is the standard deviation of the wind speed and *V̄* is the normalized wind speed.

For continuous gusts [22], we analyze the three dimensional turbulence:

(7) $$T_{i,x}=\sqrt{\frac{\Phi_{x}(\xi )\pi }{2\ell_{x}}{\left[1+{\left(\ell_{x}\xi \right)}^{2}\right]}^{2}}$$

(8) $$T_{i,y}=\sqrt{\frac{\Phi (\xi )\pi }{2\ell_{y}}\frac{{\left[1+4{\left(\ell_{y}\xi \right)}^{2}\right]}^{2}}{1+12{\left(\ell_{y}\xi \right)}^{2}}}$$

(9) $$T_{i,z}=\sqrt{\frac{\Phi_{z}(\xi )\pi }{2\ell_{z}}\frac{{\left[1+4{\left(\ell_{z}\xi \right)}^{2}\right]}^{2}}{1+12{\left(\ell_{z}\xi \right)}^{2}}}$$

where ξ is the spatial frequency, Φ is the power spectral density and ℓ is the length scale.

#### 3.1.3. Number of Rotations Analysis

The rotation rate for the anemometer [22] plays an important role in detecting faults in the nodes:

(10) $${\overset{•}{N}}_{r}=F(N_{r},\sqrt{x^{2}+y^{2}},z)$$

At steady state and constant rotation rate:

(11) F(N,V,0)=0

We obtain:

(12) $$N_{r}=N_{r}(V)$$

The relation between wind speed U and number of rotations, *N_r_* will become:

(13) $$V\propto N_{r}$$

We observe that for a specific wind speed:

(14) $$N_{rx}=N_{r,\text{rec}}$$

where:

*N_rx_* = Number of rotations for node *x*
*N_r_,_rec_* = Recorded standard value for number of rotations at the given wind speed

#### 3.1.4. Maximum Overspeeding Level

Overspeeding is another major reason for fault in anemometers.The maximum overspeeding level [20] for cup anemometer is dependent on the drag ratio and turbulence intensity:

(15) $$O_{s,\infty }=\frac{2\sqrt{h}-\sqrt{4h-{\left(1-h\right)}^{2}{\left(\frac{\Delta V}{V_{0}}\right)}^{2}}}{{\left(1-h\right)}^{2}}$$

where *h* is the drag ratio:

$$\begin{array}{l} h=\frac{C_{DL}}{C_{DH}}\\ \frac{\Delta V}{V_{0}}=T_{i} \end{array}$$

The equation becomes:

(16) $$O_{s,\infty }=\frac{2h-\sqrt{4h-{\left(1-h\right)}^{2}T_{i}^{2}}}{{\left(1-h\right)}^{2}}$$

By using second order Taylor series expansion around *T_i_* = 0, we get the simple relation:

(17) $$\begin{array}{c} O_{s,\infty }=\frac{{\left(1+h\right)}^{2}}{4h}T_{i}^{2}=\frac{T_{i}^{2}}{\left(1-\lambda_{0})(1+\lambda_{0}\right)}\\ O_{s,\infty }=F_{0}\cdot T_{i}^{2} \end{array}$$

The maximum overspeeding ratio, *F*_0_ = 1.15 at a speed ratio of 0.3, the normal speed ratio of cup anemometers [20].

Also, it is clear from above Equation (17) that:

(18) $$O_{s,\infty }\alpha T_{i}^{2}$$

which clearly shows the relation between wind speed and turbulence intensity.

### 3.2. Comparison of Anemometer with Other Technologies

**Figure 1.**
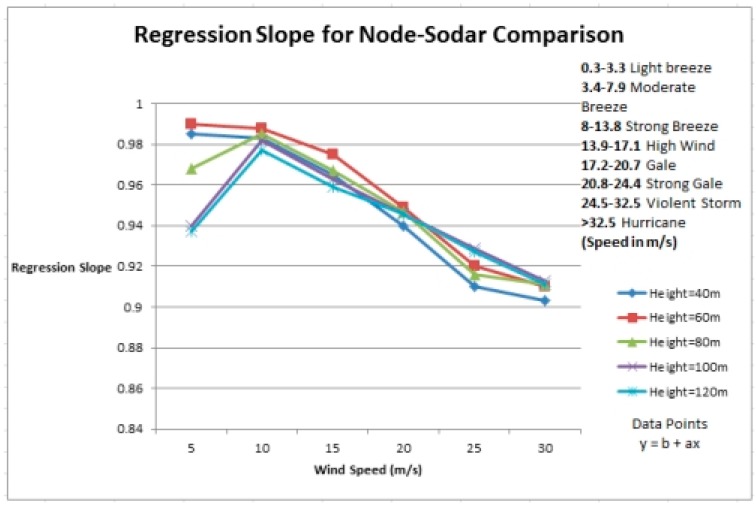
Comparison of site assessment technologies.

Before explaining our experimental analysis, we discuss the comparison of SODAR, an RS technology used for site assessment with cup anemometers. Ground-based RS techniques are also used for site evaluation nowadays. These devices can be validated by comparing the winds estimated by these devices with those recorded at the same heights by cup anemometers. Mathematical analysis was done to get Figure 1. We used our own data for anemometer while already present data for SODAR [19]. The slope is plotted using equation (19). Results show that these ground based RS devices underperform as compared to cup anemometers. In hilly terrains or areas where hindrance is sufficiently great, these devices generally record lower wind speeds [19] and are therefore suitable for use in flat terrains. The variation in the wind speed calculations performed by a node (cup anemometer) and a SODAR is shown by the regression slope in Figure 1. From the graph, it can be seen that at lower wind speeds the variation is more. However, at higher wind speeds, there appears to be a decrease in the regression slope. The range of wind speeds is according to the Beaufort scale commonly used for weather forecast by meteorological stations.

Also in Figure 1:

(19) $$\text{slope},\;\text{b}=\frac{N\sum XY-(\sum X)(\sum Y)}{\left(N\sum X^{2}-{\left(\sum X\right)}^{2}\right)}$$

and:

(20) $$\text{intercept},\;\text{a}=\frac{\sum Y-b(\sum X))}{N}$$

### 3.3. Design Details

**Figure 2.**
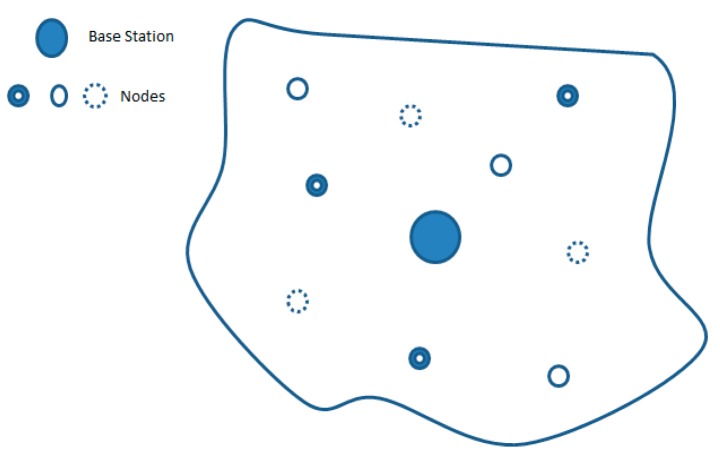
A general overview of node placement.

The design focuses on a number of anemometers deployed in a remote location which we want to assess as a potential site for deployment of wind energy project and where manual monitoring of the anemometers and fault detection becomes hard to achieve and costly. Each anemometer is equipped with a 3DR radio which operates at 433 MHz and has a range of one mile. Anemometers are therefore placed one mile apart from each other. However a large scale deployment can be done where anemometers at same heights can be placed in same clusters and can be equipped with radios that cover larger distances so that larger sites can be easily covered. For site assessment, cup anemometers are deployed in a remote location and monitoring of these anemometers is performed using a WSN. The anemometer is equipped with a 3DR as a transceiver, a memory card as a storage unit, an Arduino Uno Microcontroller [23] as a processing unit and a photo coupler as a sensing unit. The node deployment is deterministic based on same heights criteria. Nodes at same height communicate with each other for data sharing. In Figure 2, nodes placed at same height are represented by same pattern circles. Base station is deployed in the center of the assessment site while the radio range for each transceiver is 1·km. The number of nodes will increase if a vast field is to be assessed. Vast fields will require more nodes to be deployed.

## 4. Proposed Algorithm

In this section, we will explain our proposed algorithm for detecting the faulty nodes in the network.

The algorithm is divided into three parts: Algorithm 1 is for developing a monitoring system to monitor the performance of the nodes, Algorithm 2 works for detecting the faulty node and Algorithm 3 is for detecting the error due to which the fault occurred. For this purpose the task has been divided into five parts:

- Data acquisition by the nodes
- Preprocessing of the data between nodes located at same heights
- Data analysis and transmission to the sink node
- Faulty node detection at the sink node
- Data transmission to the main station via GSM

The nodes located at same height are referred to as neighbors. The nodes gather data such as wind speed, direction, number of rotations, turbulence intensity and temperature. In the second step, the node encapsulates the data with its node ID and forwards the data to its neighboring node. A node regularly shares data three times in every twenty four hours *i.e.*, nodes share data with each other once in every eight hours. However a node (cluster head) randomly decides when to share data during those eight hours. All other nodes in the cluster collect data at that time and then comparisons are made. A random variable is used in the algorithm to choose any random time during those eight hours for data sharing. The neighboring nodes share this data with each other and comparisons are made. The comparison technique is explained in Algorithm 1. If the difference between the data compared is less than a threshold percentage of 2% then the node's status is considered to be ok else the error is considered to be significant and the node transmits these results to the sink node for further analysis. Same heights criterion discussed above (Section 3.3) for getting same measurements is quite favorable in conditions like cold fronts where cold air moves rapidly to replace warmer air. In such situations, where vertical motion of air takes place, all the nodes in the same network will experience same changes in readings. Due to this the probability of false detections will be low because the algorithm relies on comparison of the readings taken by all the nodes at same height in the network.

The algorithm is divided into three parts.

- Monitoring
- Faulty Node Detection
- Fault Detection

Readings from faulty sensor nodes are geographically independent. But readings from sensor nodes in close proximity are correlated [8]. We assume that faults in the nodes do not occur regularly rather it is an event that occurs rarely. Our monitoring system is less complicated so that the nodes have to do little processing and hence save energy which will further increase the network lifetime. As the site assessment is usually expected to last for at least a year so a prolonged network lifetime would be in favor of the assessment process. In the opposite case, replacing energy drained sensors every now and then might increase the cost of the assessment project. Once a faulty node reading is detected, the results are sent to the sink node to accurately detect the error in the readings. Once a faulty node is detected, professionals can be sent to either fix the device. If the device cannot be fixed then it can either be replaced or removed from the network. We are directly working on the data collected to detect the errors in the anemometers being installed on site because the data collection process by these nodes for site assessment is carried out for months. Using this data collected, we can also find out about the node that is not giving accurate readings and can further work on correcting that node. The reason for this is that we will need that node to collect data in the coming months.



**Algorithm 1**
/* Monitoring System/* This algorithm predicts faults and sends findings to sink node**Input**: *V*: Wind speed**Input**: θ: Angle of wind incident upon the cup rotor**Input**: *N_r_*: Number of rotations**Input**: *T_i_*: Turbulence Intensity**Input**: *O_s_*: Overspeeding**Input**: τ: Torque**Input**: *t*: Temperature**Output**: monitored fault sent to sink node/* A uniform random variable x is used to share data between two nodes at random intervals/* At some random time:1Node id ← Data (*V*, θ, *N_r_, T_i_, O_s_*, τ, *t*)2Send to Node 2/* CP = comparison results of node x and (x+1) data3At Node 2:4if (CP < Threshold)5Node status = OK6end7else8if (CP > Threshold)9Send results to sink node10end


For accurate analysis at the sink node we consider an *a* × *a* squared field where N nodes are uniformly deployed. A sink node is that node in a WSN where data from all other nodes is collected. Sink node will process the data and will send it to the main station. Each node *n_i_* records measurements at regular intervals. Let *m_i_* denote the reading of the sensor node *n_i_* and *m_i_* = {*V*, θ, *N_r_, T_i_, O_s_*, τ, *t*}. Now let N (*n_i_*) denote a bounded closed set in the region that contains the node *n_i_* and additional *k* sensors *n*_1_, *n*_2_, …, *n_k_*. Let *m*_1_, *m*_2_, …, *m_k_*, denote the measurements taken by the nodes *n*_1_, *n*_2_, …, *n_k_* respectively. At the sink node comparison will be done between the reading of the node *n_i_* and the center of *m*_1_, *m*_2_, …, *m_k_*. The difference is:

(21) $$d_{i}=m_{i}-med_{m}$$

where *med_m_* denotes the median of the measurements taken. It should be clear that *d_i_* will contain not a single value, but rather multiple values as a result of the comparison of all the parameters discussed above. Similarly, we find the difference for all the sensor nodes in *N*(*n_i_*) using Equation (22) to yield *D* = {*d*_1_, *d*_2_, …, *d_i_* …, *d_k_*}. Let μ̂ and σ̂ denote the sample mean and sample standard deviation of the set *D, i.e.*,:

(22) $$\mu =\frac{1}{n}\sum_{i=1}^{n}d_{i}$$

(23) $$\hat{\sigma }=\sqrt{\frac{1}{n-1}\sum_{i=1}^{n}{\left(d_{i}-\hat{\mu }\right)}^{2}}$$

Standardize the dataset *D* to eliminate the errors and to obtain {*s*_1_, *s*_2_, …, *s_n_*}, where:

(24) $$s_{1}=\frac{d_{1}-\hat{\mu }}{\hat{\sigma }},\ldots \ldots s_{i}=\frac{d_{i}-\hat{\mu }}{\hat{\sigma }},\ldots s_{n}=\frac{d_{n}-\hat{\mu }}{\hat{\sigma }}$$

Now decision will be made based on this data.

If |*s_i_*| ≥ *Thershold*, treat *n_i_* as faulty node and assign *s_i_* to *F_d_* where *F_d_* is the set of sensors that are claimed as faulty by the above procedure.



**Algorithm 2**
/* This algorithm explains faulty node detection technique to be used at sink node.**Input**: Data (*V*, θ, *N_r_, T_i_, O_s_*, τ, *t*) from nodes**Output**: Faulty node detected1 For each node *n_i_*, construct N (*n_i_*)2 Compute *d_i_* for node *n_i_* using *N*(*n_i_*) and Equation (21)3 Compute *s_i_* for node *n_i_*4 If *s_i_* ≥ *Thershold*, treat *n_i_* as a faulty node


**Algorithm 3**
/* Fault Detection**Input**: Faulty node data**Output**: Cause of fault1for (*V_r_* = *V_r_,_rec_*)2if (*N_rx_* > *N_r_,_rec_*)3 Fault = under speeding/* Check temperature and turbulence effects4if (temperature < threshold value)5Fault = Low temperature6else7Fault = Turbulence effect8else9if (*N_rx_* < *N_r_,_rec_*)10Fault = Over speeding11Send results to main station12end


## 5. Simulation and Experimental Work

In this section, we first focus on analyzing the fault detection algorithm by simulating it using Aeolus SimWindFarm Model [24] and then implement our algorithm on our experimental setup. We then compare the simulated model with the actual experimental model to check the variation in results of simulated and implemented model and check the fault detection accuracy of the WSN. Our goal is to achieve less variation in results of experimentation and simulation results.

### 5.1. Simulation and Results

Our evaluation and results for now are based on simulation. Simulations using Matlab^®^ have been performed for obtaining the results. As mentioned before, the wind simulation and modelling is done using the Aeolus SimWindFarm toolbox [24] which is a deterministic toolbox. We implemented our algorithm on this wind farm model. Using this model we have built a network to evaluate their performance. Each simulation run follows some scenario. Each scenario is based on wind speed ranges. Wind speed ranges are given in Figure 1. All the parameters (as discussed in Section 3.1) are analyzed at these different wind speeds. The accurate analysis of these parameters will show the accuracy level of the anemometers. Ten runs are performed at each wind speed range. For example, ten runs are performed for light breeze; ten runs are performed for moderate breeze and so on. The parameters used for simulation are given in Table 1.

The faulty nodes are detected by the WSN to reduce the fault level in the results. To evaluate the performance of *F_d_*, we calculate the detection accuracy *a*(*F_d_*) and the false alarm rate, *e*(*F_d_*):

(25) $$a(F_{d})=\left|\frac{F_{d}\cap F}{F}\right|,e(F_{d})=\left|\frac{F_{d}-F}{N-\left|F\right|}\right|$$

**Figure 3.**
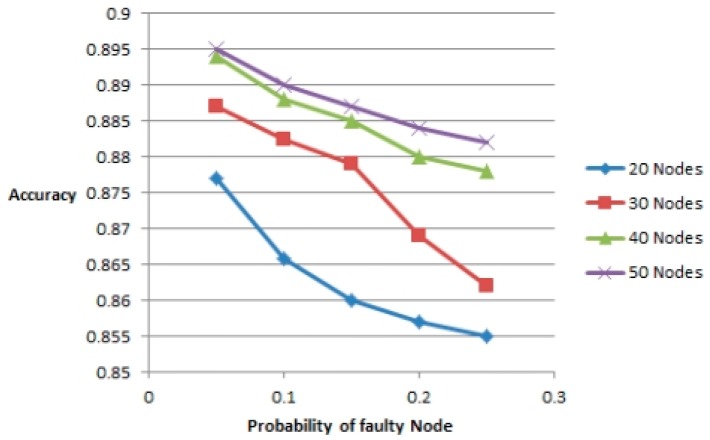
Probability of faulty node *vs.* accuracy.

**Figure 4.**
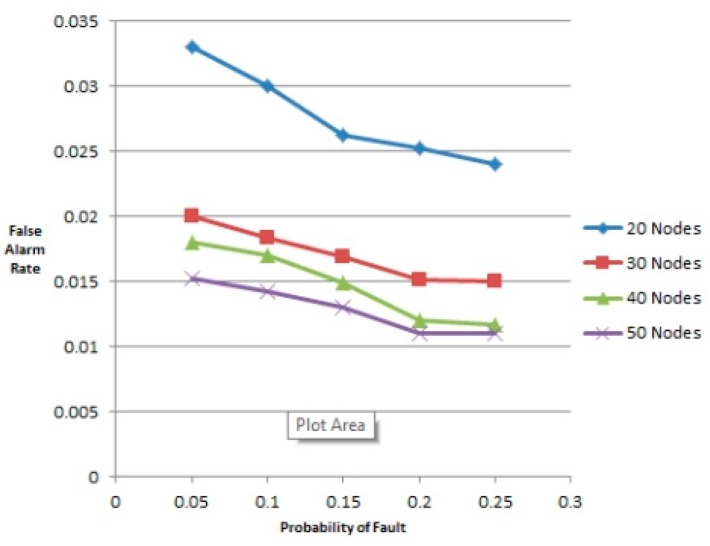
Probability of fault *vs.* false alarm rate.

If *a*(*F_d_*) is high and *e*(*F_d_*) is low, Algorithm 2 has good performance. Note that when the probability of faulty node, *p* < 0.25 and density of the network *i.e.*, the number of nodes is ≥30, then according to Figure 3, the accuracy is above 86% and in Figure 4, the false alarm rate is around 1%–2%. Increasing the number of nodes can take the accuracy level up to 89%. Although an accuracy level of 86% is achieved but it is surely not enough. The more higher the accuracy level, the more accurate assessment can be done about a site. 100% accuracy will mean that the prediction done about the energy production estimate of a particular site was 100% correct. Lower this percentage, lower will be the energy production than the estimated one. From Figure 3 it is clear that increasing the number of nodes can increase the probability of accurate assessment of a site hence taking the percentage to a much higher level. When the number of nodes increases, the accuracy level also increases. As it is a low level experimentation so we expect that a more accurate assessment can be done in future by using more nodes in the experimentation.

The results can also be improved by recording data more than three times a day. As a node's measurement is compared with the average of the recorded values so a much better average can be achieved by recording data more than once in a particular slot. For future work, it might be interesting to check the probability of getting accurate readings with increasing the number of measurements taken in a slot.

### 5.2. Experimentation

After evaluating the network using simulations, we implemented it using hardware. The anemometers were deployed in an outdoor environment along with a WSN. Each anemometer is equipped with our own designed sensor node which comprises of a photo coupler working as a sensing unit and a 3DR radio as transceiver. This WSN constantly monitors the performance of the nodes installed and sends the results to the main station via Global System for Mobile Communication (GSM). The anemometers that will share data with each other are carefully placed at the same height because anemometers at different heights will produce different readings. Our experimental analysis is a low level implementation and is therefore based on deployment of three anemometers carefully placed at the same height. We did not implement all features. To test the effectiveness of the scheme, large scale experimentation is required, involving a lot of nodes. During testing, we implemented a network with three nodes to analyze the performance through WSN. Simulation performed with fewer nodes was compared with the experimental results to compare the performance difference accurately. Data from this implemented setup is usually observed for one year. From this recorded data the annual frequency distribution of wind speed is calculated. However, we performed on site measurements for three months starting from 3 February 2014 to 15 May 2014 and worked on the results obtained. We exercised all scenarios with different wind speeds to evaluate the accuracy of the model. For wind energy related applications, the range in which the wind speed can be measured with a very high accuracy is 4–16 m/s. Below 4 m/s threshold, the wind power is almost negligible and is not worthy of consideration for the wind turbines while above 16 m/s, the power becomes almost constant and therefore can be eliminated from consideration. We consider that three nodes implementation play an important role in analyzing a potential area for wind farms, however implementation with more nodes are highly desired.

Equation (6) shows that low wind speeds usually generate higher turbulence because of the inverse relation between mean wind speed and turbulence intensity. Hence low wind speed will lead to high turbulence in the anemometers and data collected by the anemometer might have variations. A threshold value of 2% is therefore set to detect significant errors and ignore the negligible errors in the analysis of the received data. It should be noted here that if the threshold value is set below 2% then even the minor errors will be detected as major errors and the node will be declared as faulty (false positive detection) which will increase the false detection rate. If the threshold value is greater than 2% then major errors might be missed and missed detections will occur.

A node shares data packets at regular intervals with other nodes located at the same height. The following parameters are shared between the nodes:

- Wind speed, *V*
- Direction, θ
- Number of rotations, *N_r_*
- Turbulence intensity, *T_i_*
- Overspeeding, *O_s_*
- Torque, τ
- Temperature, *t*

The data shared is eventually sent to the sink node for further analysis. These parameters play an important role in detecting and correcting faults in the system. The proposed algorithm analyzes these parameters to evaluate the performance of the nodes.

A WSN is deployed to monitor the accuracy level of the anemometers and make sure that the readings taken by the anemometers are accurate. This will help in evaluating the site properly. We assume that each anemometer can compute its position through either GPS or some other techniques as mentioned in [25–27]. We use EARS routing scheme [28], a scheme which relies on radio information to route data through the network to the sink node. Cup anemometers are robust, easy to operate and are resistant to turbulence. We refer to the anemometer as a node in the remainder of the paper.

**Figure 5.**
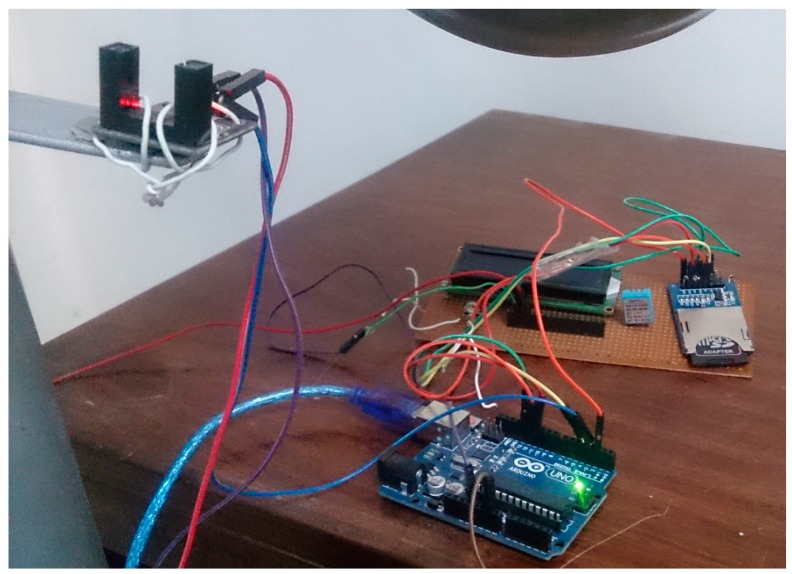
Experimental setup.

**Figure 6.**
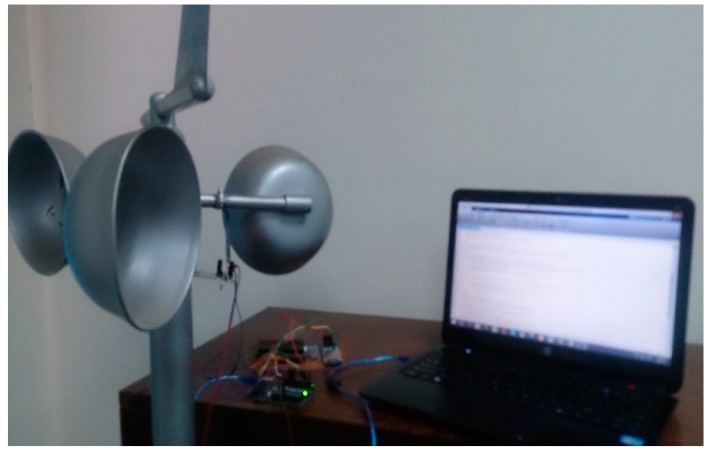
Experimental setup.

**Figure 7.**
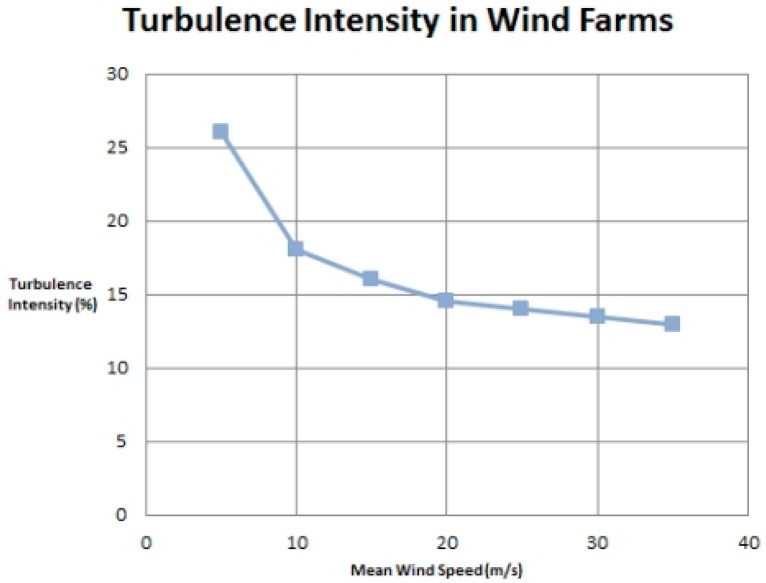
Mean wind speed *vs.* turbulence intensity.

Figures 5 and 6 show the three cup anemometer used for experiment. As discussed above, each anemometer is equipped with our own designed sensor node. Figure 5 shows the photo coupler attached to the anemometer for sensing purpose. The relation between mean wind speed and turbulence intensity according to IEC standards [29] is shown in Figure 7. It should be observed from Figure 7 that at low wind speeds, turbulence intensity is high while at higher wind speeds this intensity decreases.

### 5.3. Comparison of Simulation and Experimental Results

**Figure 8.**
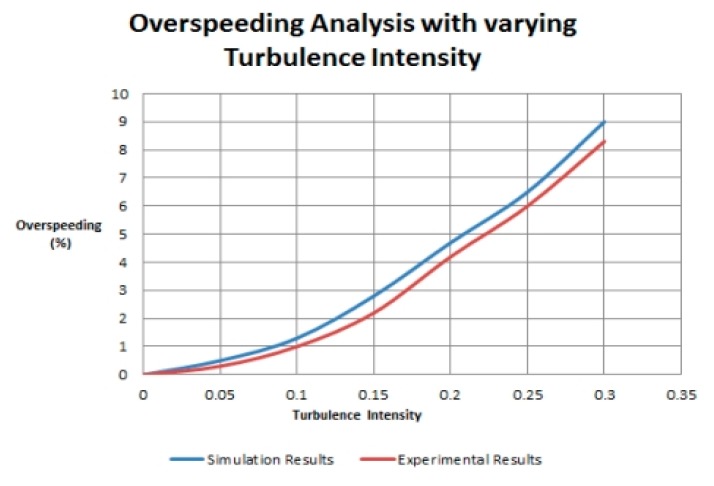
Turbulence intensity *vs.* overspeeding.

**Figure 9.**
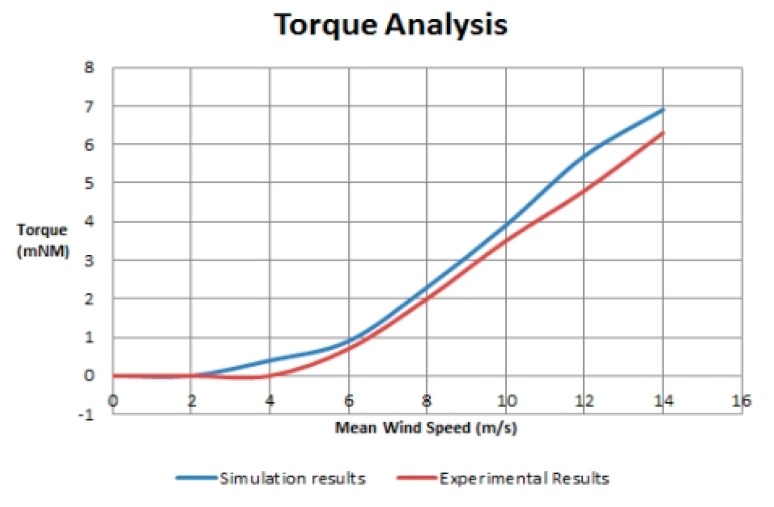
Mean wind speed *vs.* torque.

Figures 8 and 9 show the results of both simulation and experimental model. In Figure 8, overspeeding is evaluated with changing turbulence intensity while in Figure 9 torque is analyzed with changing wind speed. Both figures clearly show that actual model under performs as compared to the simulated model. Here performance refers to the level of accuracy of readings taken by the nodes. The readings refer to measurements of the parameters (discussed in Section 3.1) by the nodes. Figures 8 and 9 also show that simulation results lead to an over prediction of over speeding and torque in anemometers. Figures 8 and 9 also show that the simulation and experimental results show a maximum difference of 10%. The difference in results is basically due to the fact that in actual implementation different other factors (like radio range issues and environmental effects) are also involved which affects the performance of the model *i.e.*, the conditions are not ideal while in simulation, such issues are ignored and the conditions are ideal. The best way to deal with this issue is to consider improving the actual implemented model so that the results can be more accurate instead of working on improving the simulator. By implementing a WSN, the faults in the implemented model are detected and corrected which increases the accuracy of the implemented model. As the number of nodes in our implementation was three so the model performed properly and no faulty node was detected.

As mentioned earlier in the paper, the performance level of implemented projects is 60% of the simulated ones. We compared our simulation and experimental results and came to the conclusion that the accuracy level of implemented projects can be increased upto 86% by accurate assessment and fault detection through a network of sensor nodes. We prove the 86% accuracy level by comparing the simulation results with the experimental results.

## 6. Conclusions and Future Work

In this paper, we have proposed a technique aimed at accurate monitoring of anemometers and detection of their faults by the use of a WSN. Our simulation and experimental results show that the difference in accuracy of the simulated and actual model is around 10%–15%, which is low as compared to other implementations nowadays that show as low as 60% of the predicted performance *i.e.*, the difference is 40% between them. To the best of our knowledge this is the first paper towards accurate monitoring and fault detection in anemometers for site assessment for improved performance in implemented models.

The possible future work is to implement the proposed technique using more nodes in real hardware for the analysis of a complex network. If the experimental setup is done for a large number of nodes then work can be done on finding the probability of faulty nodes in the network. As this application is less explored, further research in this area can help the wind energy community achieve improved power performance and lower cost of wind technology and allow more widespread applications.

## Figures and Tables

**Table 1.** Simulation parameters and their values.

**Parameter**	**Value**
Field size	10 km × 10 km
Number of nodes deployed	50
Topology	Star topology with sink node and base station at the center
Radio range	1.6 km
Packet size	40 Bytes (Data = 32 Bytes + Control 8 Bytes)
Simulation time	400 s

## References

[1] Gould I.B. (2014). Wind Energy Deployment in Isolated Islanded Power Systems: Challenges and Realities.

[2] Dahlberg J.A., Gustavsson J., Ronsten G., Pedersen T.F., Paulsen U.S., Westermann D., Helm P., Zervos A. (2001). Development of a Standardised Cup Anemometer Suited to Wind Energy Applications, Classcup. Wind Energy for the New Millennium.

[3] Kristensen L., Jensen G., Hansen A., Kirkegaard P. (2001). Field Calibration of Cup Anemometers.

[4] World Wind Energy Association World Wind Energy Report 2008.

[5] Staddon J., Balfanz D., Durfee G. Efficient Tracing of Failed Nodes in Sensor Networks.

[6] Chintalapudi K.K., Govindan R. (2003). Localized Edge Detection in Sensor Fields. Ad Hoc Netw..

[7] Clouqueur T., Saluja K.K., Ramanathan P. (2004). Fault Tolerance in Collaborative Sensor Networks for Target Detection. IEEE Trans. Comput..

[8] Krishnamachari B., Iyengar S. (2004). Distributed Bayesian Algorithms for Fault-Tolerant Event Region Detection in Wireless Sensor Networks. IEEE Trans. Comput..

[9] Zaher A., McArthur S.D.J., Infield D.G., Patel Y. (2009). Online wind turbine fault detection through automated SCADA data analysis. Wind Energy.

[10] Hameed Z., Hong Y.S., Cho Y.M., Ahn S.H., Song C.K. (2009). Condition Monitoring and Fault Detection of Wind Turbines and Related Algorithms: A Review. Renew. Sustain. Energy Rev..

[11] Ingelrest F., Barrenetxea G., Schaefer G., Vetterli M., Couach O., Parlange M. (2010). SensorScope: Application-specific sensor network for environmental monitoring. ACM Trans. Sens. Netw..

[12] Weimer M.A., Paing T.S., Zane R.A. Remote Area Wind Energy Harvesting for Low-Power Autonomous Sensors.

[13] Melcher J.R. (1981). Continuum Electromechanics.

[14] Teager H. (1980). Some Observations on Oral Air Flow during Phonation. IEEE Trans. Acoust. Speech Signal Process.

[15] Andy S. (2009). A Measurement Breeze. Quality Manufacturing Today.

[16] Haaren R.V., Fthenakis V. (2011). GIS-based wind farm site selection using spatial multi-criteria analysis (SMCA): Evaluating the case for New York State. Renew. Sustain. Energy Rev..

[17] Longatt F.G., Gonzalez J.S., Payan M.B., Santos J.M.R. (2014). Wind resource atlas of Venezuela based on on-site anemometry observation. Renew. Sustain. Energy Rev..

[18] Wharton S., Qualley G., Newman J., Miller W. (2013). LIDAR Campaign at Buena Vista Wind Farm: An Examination of Hill Speedup Flows.

[19] Steven L., Eamon M. (2011). LIDAR and SODAR Measurements of Wind Speed and Direction in Upland Terrain for Wind Energy Purposes. Remote Sens..

[20] Pedersen T.F. (2003). Development of a Classification System for Cup Anemometers—CLASSCUP.

[21] International Electrotechnical Commission (IEC) (2005). Wind Turbines—Part 12–1: Power Performance Measurements of Electricity Producing Wind Turbines.

[22] Kristensen L. (1993). The Cup Anemometer and Other Exciting Instruments.

[23] Arduino Uno. http://arduino.cc/en/Main/arduinoBoardUno.

[24] Aeolus SimWindFarm. http://www.ict-aeolus.eu/SimWindFarm/.

[25] Tariq M., Kim Y.P., Kim J.H., Park Y.J. Energy Efficient and Reliable Routing Scheme for Wireless Sensor Networks.

[26] Cheng X., Thaeler A., Xue G., Chen D. TPS: A Time-Based Positioning Scheme for Outdoor Sensor Networks.

[27] Ruml W., Shang Y., Zhang Y. Location from Mere Connectivity.

[28] Wu H., Wang C., Tzeng N.F. (2005). Novel Self-Configurable Positioning Technique for Multi-Hop Wireless Networks. IEEE ACM Trans. Netw..

[29] (1998). Wind Turbine Generator Systems-Part 1: Safety Requirements.

